# Efficacy of mobile applications in treating depression: systemic review and meta-analysis

**DOI:** 10.1192/bjb.2025.10119

**Published:** 2026-04

**Authors:** Eiman Araib, Usama Khan, Umama Alam, Manahil Moazzam

**Affiliations:** 1 Internal Medicine, Dow University of Health Sciences, Karachi, Pakistan; 2 Internal Medicine, Nowshera Medical College, Nowshera, Pakistan; 3 Internal Medicine, Khyber Medical College, Peshawar, Pakistan

**Keywords:** Meta-analysis, depressive disorders, evidence-based mental health, experiment design, quality of life

## Abstract

**Aims and method:**

Previous meta-analysis of the efficacy of mobile phone applications (mHealth apps) for depression has several limitations, including high risk of bias and heterogeneity in effect sizes across studies, and gaps in understanding of variability in treatment outcomes. We aimed to provide more reliable and clinically relevant findings by conducting a systematic literature search on PubMed, Embase and PsycInfo, focusing on newer studies with minimal risk of bias.

**Results:**

Analysing 17 randomised controlled trials (*n* = 2821) published between 2020 and 2025, we found a pooled standardised mean difference (s.m.d.) of –0.46 (95% CI –0.64 to –0.28; *P* < 0.001) relative to the control groups, which indicates a significant reduction in depressive symptoms. Subgroup analyses confirmed efficacy in both adolescents (s.m.d. = –0.42) and adults (s.m.d. = –0.49). Despite evidence of publication bias, 70% of the studies had a low risk of bias, supporting the robustness and reliability of these findings.

**Clinical implications:**

The results underscore the clinical relevance of mHealth apps as scalable and accessible tools for bridging gaps in mental healthcare. Their effectiveness across age groups highlights their potential for broad implementation, with future research needed to refine personalisation, engagement strategies and methodological rigour.

Depression is a serious and debilitating mental health condition that affects millions of people worldwide. It is characterised by persistent feelings of sadness, a loss of interest or pleasure in activities and difficulties in performing daily tasks. This condition is one of the leading causes of disability globally, with an estimated lifetime prevalence of 20% and an annual prevalence of 5–10% in high-income countries.^1,2^ According to the World Health Organization (WHO), depression is a major contributor to the global burden of disease, with prevalence rates continuing to rise among various groups, including adolescents, university students and working-age adults.^1^ Effective treatments are available, such as medications and psychotherapy, but many people face significant barriers to accessing care. These barriers include the stigma associated with mental health problems, the high cost of treatment and the limited availability of healthcare resources in many regions.^3^ Such challenges emphasise the urgent need for innovative and scalable solutions that can make mental healthcare more accessible to those in need.

One such promising solution is mobile health (mHealth) applications (apps), which leverage the widespread use of smartphones to deliver evidence-based treatments for depression. These apps often incorporate therapeutic approaches such as cognitive–behavioural therapy (CBT), behavioural activation and mindfulness techniques to help users manage and reduce their depressive symptoms.^4,5^ Mobile apps offer several advantages, including being cost-effective, scalable and easily accessible. This makes them particularly valuable for populations that face challenges accessing traditional mental health services. Studies have shown that these apps can effectively reduce symptoms of depression and improve mental well-being in a variety of groups.^6,7^ In recent years, randomised controlled trials (RCTs) have further demonstrated the effectiveness of specific mHealth apps. For example, the Feel Stress Free app, which integrates CBT-based modules with features like mood tracking and relaxation exercises, has been shown to significantly reduce depressive symptoms among university students.^8^ Similarly, the SPARX app, which is designed for adolescents, has shown promising results in managing mild to severe depressive symptoms through self-guided CBT.^9^ Additionally, the HeadGear app has proven effective in workplace settings, helping to prevent the onset of depression among employees.^10^ These findings highlight the potential of mHealth interventions to address the diverse needs of individuals across different settings.

The systematic review and meta-analysis conducted by Bae et al in 2023 offered valuable insights into the effectiveness of mHealth interventions for moderate to severe depression. It reported a medium effect size and identified factors such as app design, intervention duration and population characteristics that influenced treatment outcomes.^11^ However, this analysis also revealed some important gaps in the current understanding of the efficacy of mHealth interventions. For instance, there was considerable heterogeneity in effect sizes across studies, which was likely due to differences in app features and the characteristics of study populations. Furthermore, concerns were raised about the variability in methodological quality, with some studies exhibiting higher risk of bias. Another key limitation was the lack of detailed exploration into how moderating factors interact to influence outcomes. Finally, the analysis only included data from studies published up to early 2023, meaning that newer RCTs were not considered.^11^

To address these limitations and reach a conclusion this updated meta-analysis incorporates additional RCTs to reassess the efficacy of mobile apps in treating depression. By focusing on studies with minimal risk of bias and analysing newer data, this study aims to provide more reliable and clinically relevant findings. Additionally, this analysis seeks to identify and understand the sources of variability in treatment outcomes, such as differences in app design, intervention delivery and population characteristics. By doing so, this updated meta-analysis hopes to contribute to the development and refinement of mHealth solutions, ensuring their successful integration into mainstream mental healthcare. Ultimately, these findings aim to improve access to effective treatments and enhance mental health outcomes for individuals suffering from depression.

## Method

### Search strategy

We conducted a systematic literature search on PubMed, Embase and PsycInfo from inception to 10 January 2025, using the participants, intervention, comparison, outcome and study design (PICOS) framework and Medical Subject Heading (MeSH) terms for depression, mobile applications and randomised clinical trials. This meta-analysis followed PRISMA guidelines and included 17 studies, combining 13 studies identified in the previous meta-analysis^11^ and 4 newly added RCTs,^4–6,12^ assessing the effectiveness of app-based psychological interventions for reducing depressive symptoms.

### Inclusion and exclusion criteria

Studies were eligible if they were RCTs, published between 2020 and 2025, included participants aged 18 years or older, used validated depression scales (such as the PHQ-8, PHQ-9, DASS-21R or HADS) and delivered interventions via mobile apps employing evidence-based approaches such as CBT, behavioural activation, mindfulness, cognitive bias modification and rational emotive behaviour therapy. We equated the different depression scales using Huan et al’s^13^ scaling method. Comparison groups had to include waiting-list controls, psychoeducational apps or active placebo conditions. Additionally, studies were required to report sufficient data for calculating effect sizes.

Studies were excluded if they combined app-based interventions with pharmacological treatments, had participants with baseline depression scores below the clinical threshold (PHQ-9 equivalent of 0–3) or were non-randomised in design. Other exclusion criteria included lack of a control group, incomplete or missing post-intervention outcome data, or a primary focus on mental health conditions other than depression. These criteria were designed to ensure the inclusion of methodologically sound and clinically relevant studies for meta-analytic synthesis.

### Study selection and data extraction

**Fig. 1:**
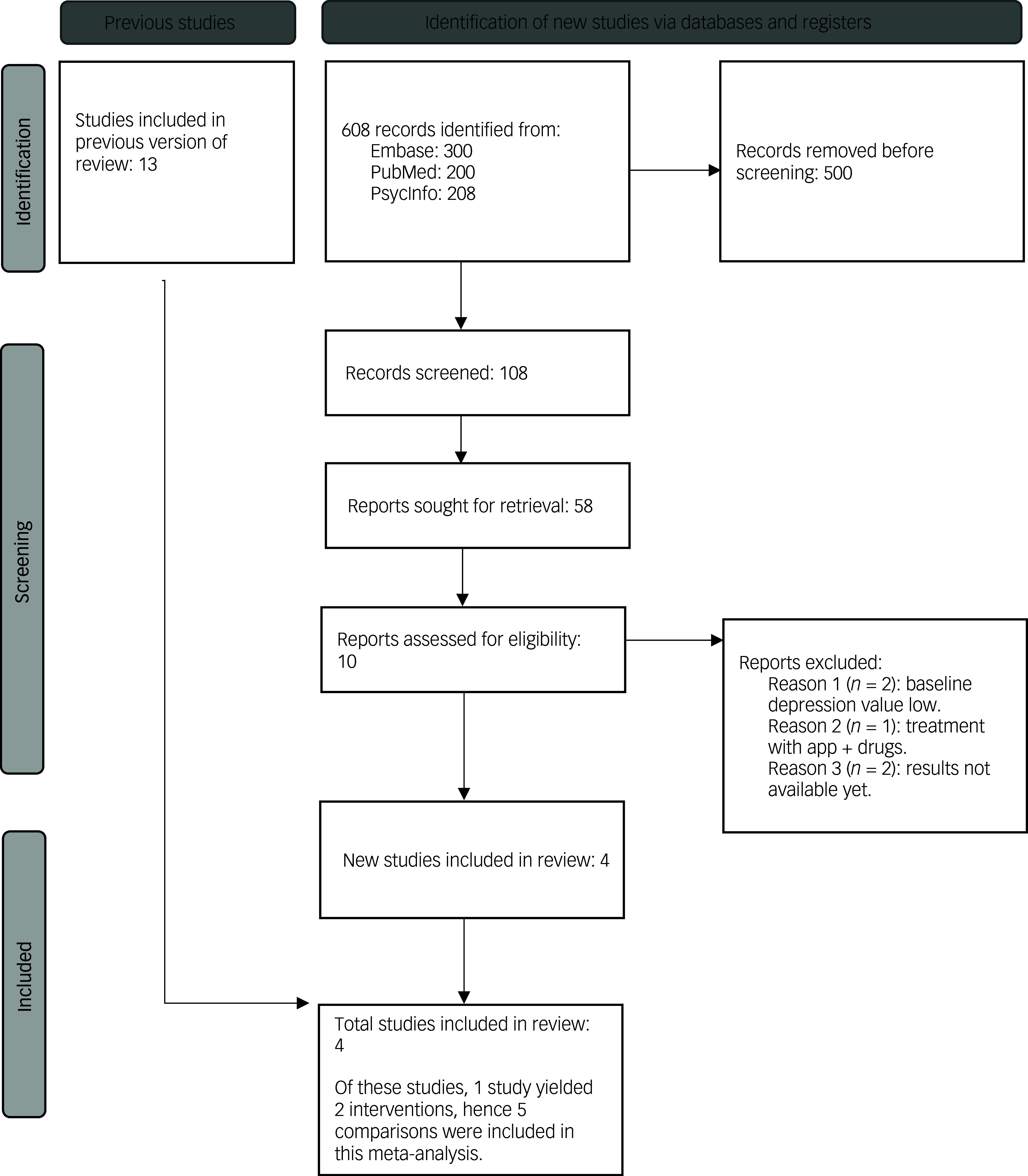
PRISMA flow diagram showing study selection process. app, mobile phone application.

Two researchers independently conducted the literature search and data extraction, with a third researcher resolving any disagreements through discussion. The meta-analysis employed a systematic approach to study selection and data extraction. The search yielded 608 records, 500 of which were excluded because of duplication or irrelevance. After screening the remaining 108 articles, 58 underwent full-text review: 10 studies met the inclusion criteria, including 4 newly added studies (Fig. 1). One study contributed two interventions and five comparisons. Key variables extracted included study characteristics, participant demographics, intervention details, comparison groups and the primary outcome measure, which was the standardised mean difference (s.m.d.) in depressive symptom reduction. Secondary outcomes, such as drop-out rates and intervention durations, were also included to ensure comprehensive data analysis.

### Quality assessment

The quality of included studies was evaluated using the Cochrane Risk of Bias Tool (RoB 2) against five bias domains: (a) bias arising from the randomisation process; (b) bias due to deviations from intended interventions; (c) bias due to missing outcome data; (d) bias in the measurement of the outcome; and (e) bias in the selection of the reported result. Final judgements were established by consensus. We applied two different strategies to evaluate the quality of the literature, and this evaluation was accomplished by two researchers. RoB 2 was applied to RCTs and the Newcastle–Ottawa Scale (NOS) was used for cohort studies. The assessment revealed that 70% of the studies had a low risk of bias, 25% had some concerns and 5% were rated as having a high risk of bias. The main problems identified were related to deviations from the intended interventions and outcome measurement. This thorough evaluation ensured the methodological rigour of the included studies and highlighted the overall reliability of the findings.

### Statistical analysis

We evaluated the heterogeneity of the combined data using a systematic approach. The *I* ^2^ statistic was used to quantify heterogeneity, with thresholds defined as follows: *I*² ≥ 75% indicated high heterogeneity, 50% ≤ *I*² < 75% represented moderate heterogeneity, and 25% ≤ *I*² < 50% indicated low heterogeneity. If *I*² was 0, a fixed-effects model was applied; otherwise, a random-effects model was used. For cases of heterogeneity, sensitivity or subgroup analyses were conducted to identify its potential sources. The *I*² value for this analysis was 81%, indicating substantial heterogeneity, with a *τ*² value of 0.11. The primary outcome, the standardised mean difference (s.m.d.) in depressive symptom reduction, was synthesised alongside secondary outcomes, including drop-out rates and intervention durations. Funnel plot asymmetry was used to assess publication bias across studies. These statistical methods ensured a thorough and reliable synthesis of the data while addressing variability and potential biases.

## Results

This meta-analysis included 4 new studies (from the UK,^4^ USA,^6^ Australia^5^ and Romania^12^) (Table 1) and 13 studies from a previous meta-analysis; together, the 17 studies involved 2821 participants with mild to severe depression.

**Table 1:** Characteristics of the studies included in the meta-analysis

Author	Year	Country	Study design	Sample size, *n*		Age, years: mean (s.d.)	Intervention	Control	Baseline depression score, mean (s.d.)	Study duration, weeks	Drop-out rate, %
				Intervention	Control						
McCloud et al^4^	2020	UK	Web-based, parallel group, unmasked RCT	84	84	≥18	Feel Stress Free app (CBT-based)	Waiting list	Scored ≥8 on one or both subscales of the HADS	6	34
Peake et al^6^	2024	USA	Open-label RCT	74	79	16 (2.5)	Spark app (CBT-based digital therapeutics)	Psychoeducational app	Mild to severe depression cohort PHQ-8 score: Spark: 14.36 (4.78) Control: 13.29 (4.51)	5	24.4
Deady et al^5^	2024	Australia	RCT	1128	1143	40	HeadGear app (behavioural activation and mindfulness)	Mood-monitoring app	PHQ-9 scores of 6.9 were measured	Assessment at 5, 12 and 52 weeks	54% at 3 months, 78.5–83.7% at 12 months
Sîrbu et al^12^	2025	Romania	Multi-arm, parallel-group, randomised trial	PsyPills: 80 OCAT: 70	79	30 (10.7)	PsyPills app (REBT-based)OCAT app (CBM-based)	Sham OCAT (active placebo)	DASS-21R score: PsyPills 4.31 OCAT: 5.41 Sham OCAT: 5.13	4	47% at mid, 59.83% at post and 71.18% at 1 month follow-up

app, application; CBM, cognitive bias modification; CBT, cognitive–behavioural therapy; HADS, Hospital Anxiety and Depression Scale; OCAT, online contingent attention training; PHQ, Patient Health Questionnaire; RCT, randomised controlled trial; REBT, rational emotive behaviour therapy; DASS, Depression Anxiety Stress Scales.

Pooled results for the 17 studies^4–6,11,12^ showed a reduction in depressive symptoms with the use of mobile apps relative to the control groups: s.m.d. = −0.46; 95% CI −0.64 to −0.28; *P* < 0.001; *I*² = 81% (the forest plot can be seen in Supplementary Fig. 1, available online at https://doi.org/10.1192/bjb.2025.10119). Pooled results for the four new studies and the seven studies with minimum risk of bias from the previous meta-analysis showed similar reduction in symptoms: s.m.d. = −0.45; 95% CI −0.66 to −0.24; *P* < 0.001; *I*² = 79% (Supplementary Fig. 2). In both analyses, these values are statistically significant.

We conducted a subgroup analysis for adolescents only, as described by Sawyer et al,^14^ and concluded that adolescents using mobile apps also showed a reduction in depressive symptoms compared with the control groups: s.m.d. = −0.42; 95% CI, −0.7 to −0.14; *P* < 0.001; *I*² = 73% (Supplementary Fig. 3). A subgroup analysis for adults showed a similar reduction: s.m.d. = −0.49; 95% CI −0.72 to −0.26; *P* < 0.001; *I*² = 82% (Supplementary Fig. 4). These values are again statistically significant.

### Risk of bias assessment

The risk of bias assessment for the four new studies included in our meta-analysis was conducted using the Cochrane Risk of Bias Tool (RoB 2). The lowest risk of bias was observed in the randomisation process. Bias due to missing outcome data and selection of reported results was determined to be low. However, concerns were identified regarding bias arising from deviations from the intended interventions and outcome measurement. Overall, the assessment indicated that 70% of the studies had a low risk of bias, 25% had some concerns and 5% had a high risk of bias (Supplementary Fig. 9). The traffic light plot for all included studies is shown in Supplementary Fig. 10. Additionally, domain-specific concerns were noted, particularly regarding measurement and reporting biases.

## Discussion

The use of mobile apps for mental health has grown exponentially in recent years, offering scalable and accessible solutions to address the global burden of depression. This updated meta-analysis, incorporating four recent RCTs, provides a comprehensive evaluation of their efficacy in reducing symptoms of depression. Previous meta-analyses have consistently highlighted the potential of these digital interventions, and the current analysis sheds light on the variability in outcomes across different populations and app designs. By integrating data from a diverse range of studies, this discussion aims to interpret the findings in the context of existing literature, evaluate the strengths and limitations of the analysis, and outline practical implications and future research directions to optimise the use of mobile apps in mental healthcare.

### Interpretation of findings

The results of this updated meta-analysis confirm moderate efficacy for mobile apps in reducing depression symptoms, with a pooled standardised mean difference (s.m.d.) of −0.46 (95% CI −0.64 to −0.28). However, the substantial heterogeneity observed (*I*² = 81%) necessitates careful consideration of the factors contributing to variability. The high heterogeneity can be attributed to differences in study populations, intervention designs and levels of bias across the included studies.

The risk of bias assessment provides valuable insights into these discrepancies. Although 70% of studies were rated as low risk overall, some concerns were identified in domains such as deviations from intended interventions (RoB 2: domain 2) and measurement of outcomes (domain 4). Notably, Mantani et al^15^ had a high risk of bias due to problems with outcome measurement, which may have influenced their results. Similarly, Tønning et al^16^ and Raevuori et al^17^ demonstrated concerns in the domain of deviations from intended interventions, reflecting potential inconsistencies in intervention delivery.

The variability in effect sizes across subgroups aligns with these bias concerns. For example, studies such as Peake et al,^6^ which had low overall risk of bias, demonstrated consistent but modest effects (−0.1679) as shown in Supplementary Fig. 2 for a mobile app for adolescents, whereas interventions targeting broader populations (e.g. Sîrbu et al^12^) exhibited larger effects but also higher heterogeneity due to variations in adherence and implementation. This heterogeneity underscores the influence of methodological rigour and study-specific factors on pooled outcomes. The heterogeneity in our analysis of the 4 new studies and all 13 studies from the previous meta-analysis was 81%. After removing high risk of bias studies it decreased slightly, to 79%. Our subgroup analyses of adolescents decreased it to 73%.

In summary, although the findings reaffirm the moderate efficacy of mobile apps for depression, the observed heterogeneity and risk of bias highlight the need for standardised methodologies and rigorous intervention designs in future research. These factors are crucial for improving the reliability and applicability of evidence in this field.

### Comparison with existing literature

The efficacy of app-based interventions for depression has been the subject of numerous studies, with varying outcomes across different populations and intervention designs. Our meta-analysis, which included a subgroup analysis of adults and adolescents, found a standardised mean difference of −0.46 (95% CI −0.64 to −0.28) in depressive symptom reduction, indicating moderate effectiveness. This aligns with previous research, such as the systematic review and meta-analysis by Bae et al,^11^ which reported a medium effect size for app-based interventions targeting moderate to severe depression.

When examining age-specific outcomes, our subgroup analysis revealed an s.m.d. of −0.42 (95% CI −0.70 to −0.14) for adolescents and −0.49 (95% CI −0.72 to −0.26) for adults, both statistically significant. These findings are consistent with a meta-analysis by Noh et al,^18^ which demonstrated significant effects of internet-based CBT on depression in adolescents and young adults.

However, it is important to note the substantial heterogeneity observed in our analyses (*I*² = 81%), which suggests variability in intervention effectiveness across studies. This heterogeneity may stem from differences in app features, intervention durations and participant characteristics. For instance, a systematic review by Firth et al^19^ highlighted that app-based interventions incorporating elements such as mood tracking and personalised feedback tend to yield more substantial reductions in depressive symptoms.

Our findings corroborate existing literature supporting the moderate efficacy of app-based interventions for depression across diverse populations. The observed heterogeneity underscores the need for future research to identify and address factors contributing to variability in intervention outcomes.

### Strengths and limitations

This meta-analysis has several strengths. The inclusion of both new and previously analysed studies allows for a robust evaluation of mobile app efficacy. The broad demographic scope enhances the generalisability of findings, and the detailed assessment of risk of bias ensures a high level of methodological rigour. Additionally, the use of standardised outcome measures across studies strengthens the reliability of the pooled estimates.

However, there are limitations. High heterogeneity indicates that the findings should be interpreted cautiously, as the variability in app design, study populations and comparator groups might affect the generalisability of results. Some studies, such as Sîrbu et al,^12^ reported low adherence rates, potentially skewing their effect sizes. Moreover, publication bias, as suggested by the funnel plot asymmetry (shown in Supplementary Fig. 5 for all studies and Supplementary Fig. 6 for minimum risk of bias studies), may have led to overestimation of the pooled effect size. However, the subgroup analysis for adolescents (Supplementary Fig. 7) and adults (Supplementary Fig. 8) shows a relatively symmetrical funnel plot. Lastly, the short follow-up periods in many studies hinder our understanding of the long-term efficacy of these interventions.

### Implications for practice, policy and future research

The findings of this meta-analysis have important implications for clinical practice and policy. Mobile apps can serve as scalable and accessible tools to address the global burden of depression, particularly in settings with limited mental health resources. Policymakers should prioritise integrating evidence-based mHealth interventions into public health frameworks to enhance access to mental healthcare. A systematic review found that app-based interventions led to significant improvements in depression and stress symptoms across various outcomes, demonstrating their effectiveness in diverse settings.^20^ However, further research is needed to explore the sustainability of these improvements and better understand the factors contributing to variability in their effectiveness. Specifically, studies should explore the long-term sustainability of app-based interventions and their effectiveness across diverse cultural and socioeconomic contexts. Greater attention to user engagement strategies, such as gamification and personalisation, could improve adherence and outcomes, particularly in younger populations. A systematic review emphasised the importance of examining both objective and subjective engagement in mHealth interventions for depression, noting that engagement levels can significantly influence outcomes.^21^ Additionally, rigorous head-to-head comparisons of different app designs, such as those based on CBT versus cognitive bias modification, could provide insights into the most effective therapeutic mechanisms.^22^ Ongoing studies like Beintner et al^23^ could provide more data on the efficacy of using apps for treating depression.

## Supporting information

Araib et al. supplementary material Araib et al. supplementary material

## Data Availability

Data availability is not applicable to this article as no new data were created or analysed in this study.

## About the authors

**Eiman Araib**, Medical Officer, Internal Medicine, Dow University of Health Sciences, Karachi, Pakistan. **Usama Khan**, Medical Graduate, Internal Medicine, Nowshera Medical College, Nowshera, Pakistan. **Umama Alam**, Medical Graduate, Internal Medicine, Khyber Medical College, Peshawar, Pakistan. **Manahil Moazzam**, Medical Graduate, Internal Medicine, Khyber Medical College, Peshawar, Pakistan.
