# Supplementary material for: Efficacy of mobile applications in treating depression: systemic review and meta-analysis
Source: BJPsych Bull. 2025 Jul 10;50(2):156–62. doi: 10.1192/bjb.2025.10119 (PMC13150495; doi:10.1192/bjb.2025.10119)
Supplement: Araib et al. supplementary material [file S2056469425101198sup001.docx]

# SUPPLEMENTARY FILE

**Search string**

(("app"[All Fields]) AND ("based"[All Fields] OR "basing"[All Fields]) AND ("intervention s"[All Fields] OR "interventions"[All Fields] OR "interventive"[All Fields] OR "methods"[MeSH Terms] OR "methods"[All Fields] OR "intervention"[All Fields] OR "interventional"[All Fields]) AND ("depressed"[All Fields] OR "depression"[MeSH Terms] OR "depression"[All Fields] OR "depressions"[All Fields] OR "depression s"[All Fields] OR "depressive disorder"[MeSH Terms] OR ("depressive"[All Fields] AND "disorder"[All Fields]) OR "depressive disorder"[All Fields] OR "depressivity"[All Fields] OR "depressive"[All Fields] OR "depressively"[All Fields] OR "depressiveness"[All Fields] OR "depressives"[All Fields])) AND (randomizedcontrolledtrial[Filter])

**PICO**

P: adults (age >= 18), having mild to severe depression

I: Apps used to treat depression

C: Both active controls and inactive controls

O: Measure depression levels

**Forest Plots**

**
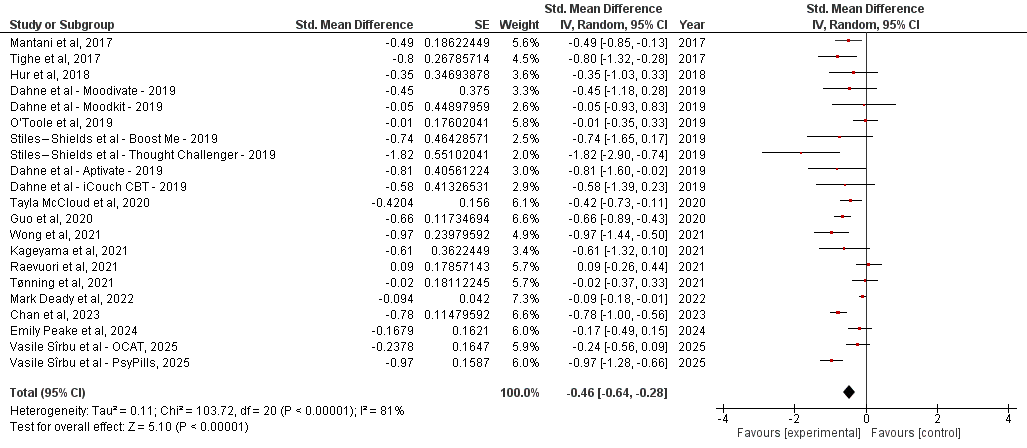
**

*Supp-Figure 1: 4 new RCTs and 13 previous RCTs forest plot*

**
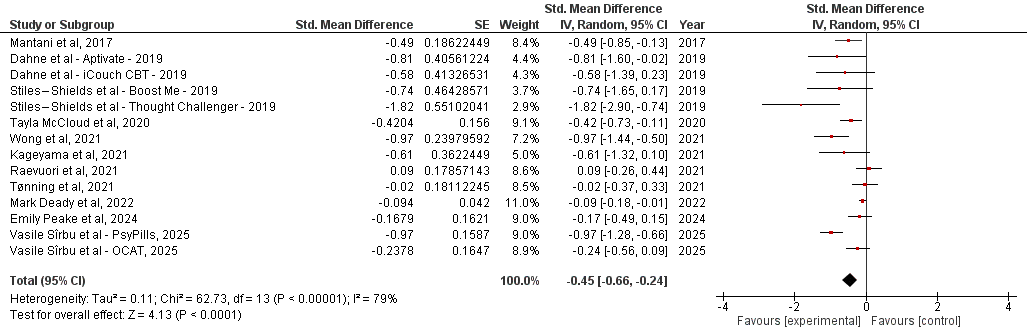
**

*Supp-Figure 2: 4 new RCTs and 7 previous RCTs with minimum RoB forest plot*

**
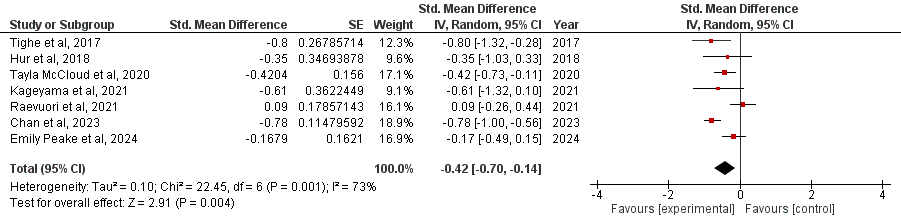
**

*Supp-Figure 3: Forest plot for adolescent*

**
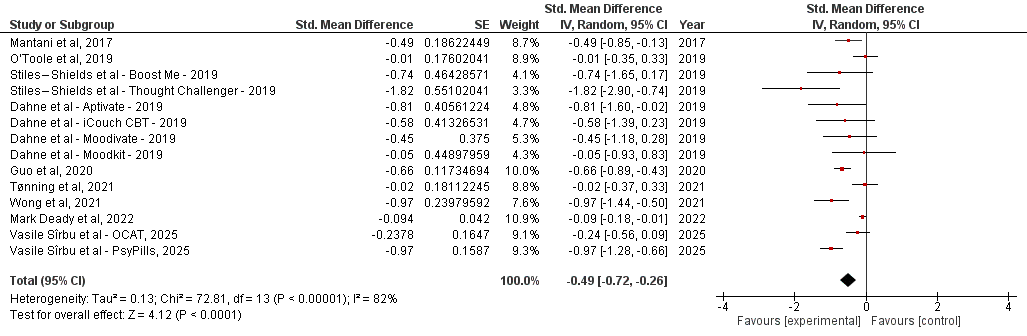
**

*Supp-Figure 4: Forest plot for adults*

**Funnel Plots**

**
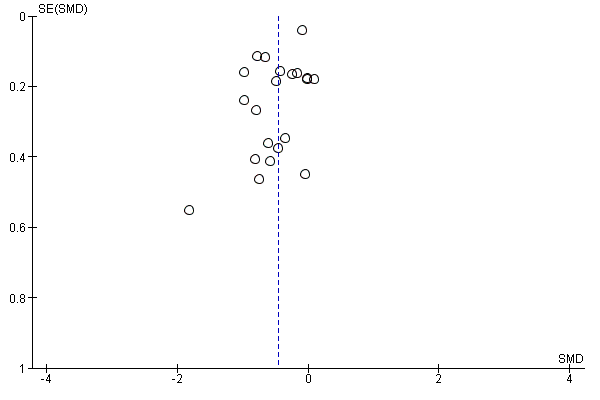
**

*Supp-Figure 5: 4 new RCTs and 13 previous RCTs funnel plot*

**
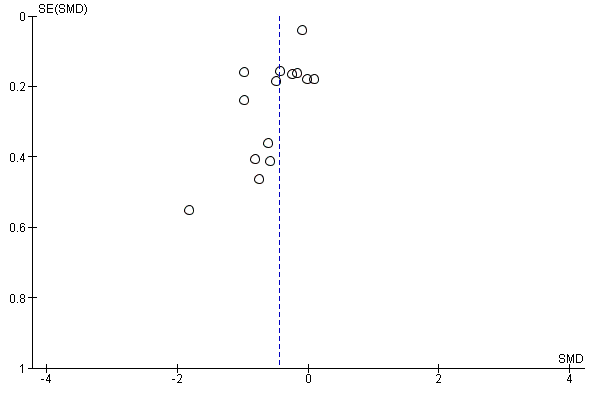
**

*Supp-Figure 6: 4 new RCTs and 7 previous RCTs with minimum RoB funnel plot*

**
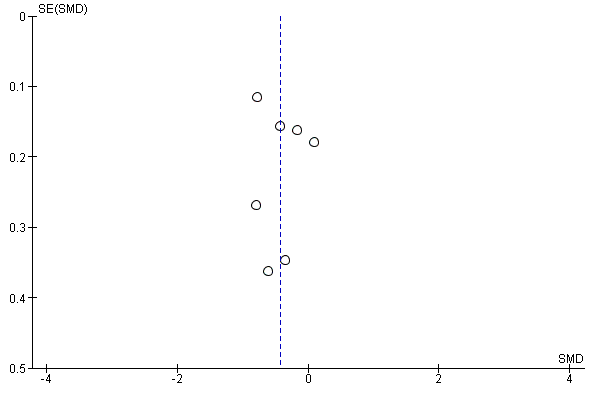
**

*Supp-Figure 7: Funnel plot for adolescent*

**
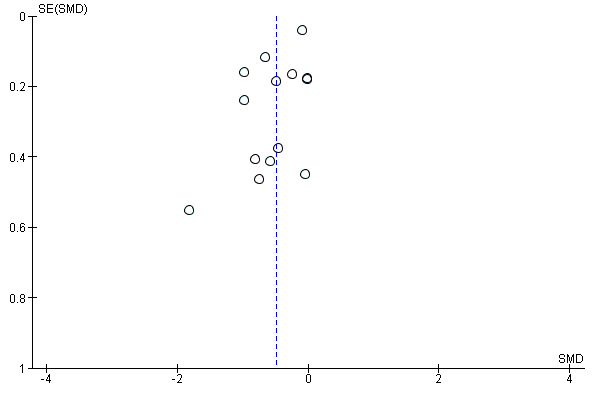
**

*Supp-Figure 8: Funnel plot for adults*

**Risk of Bias Analysis**

**
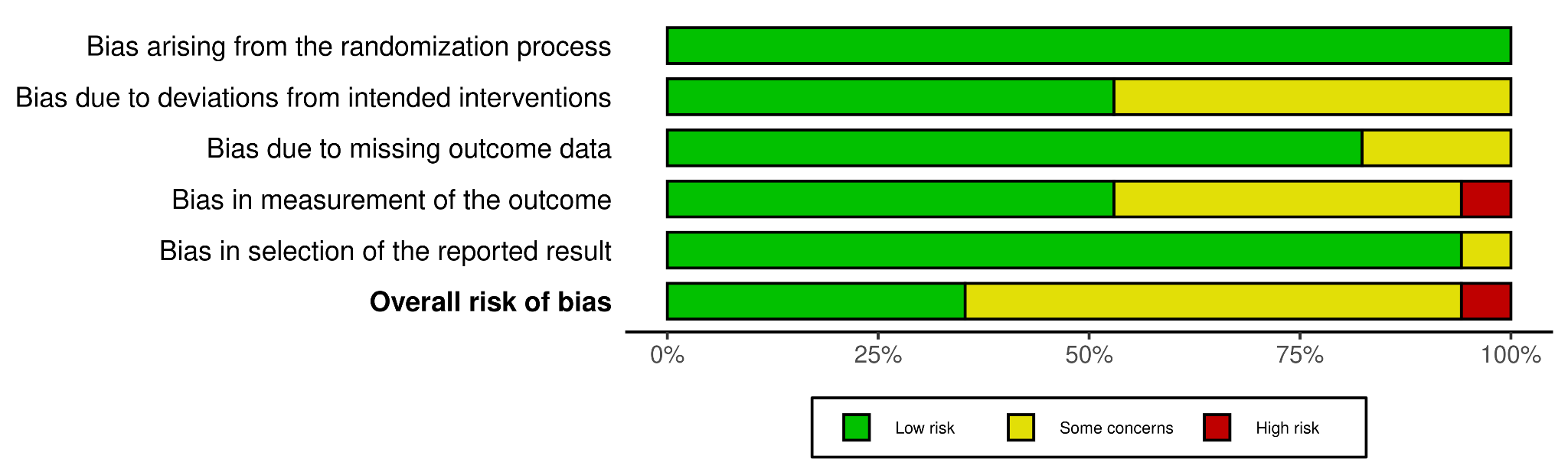
**

*Supp-Figure 9: The Overall Risk of Bias Summary of the Included Studies*

**
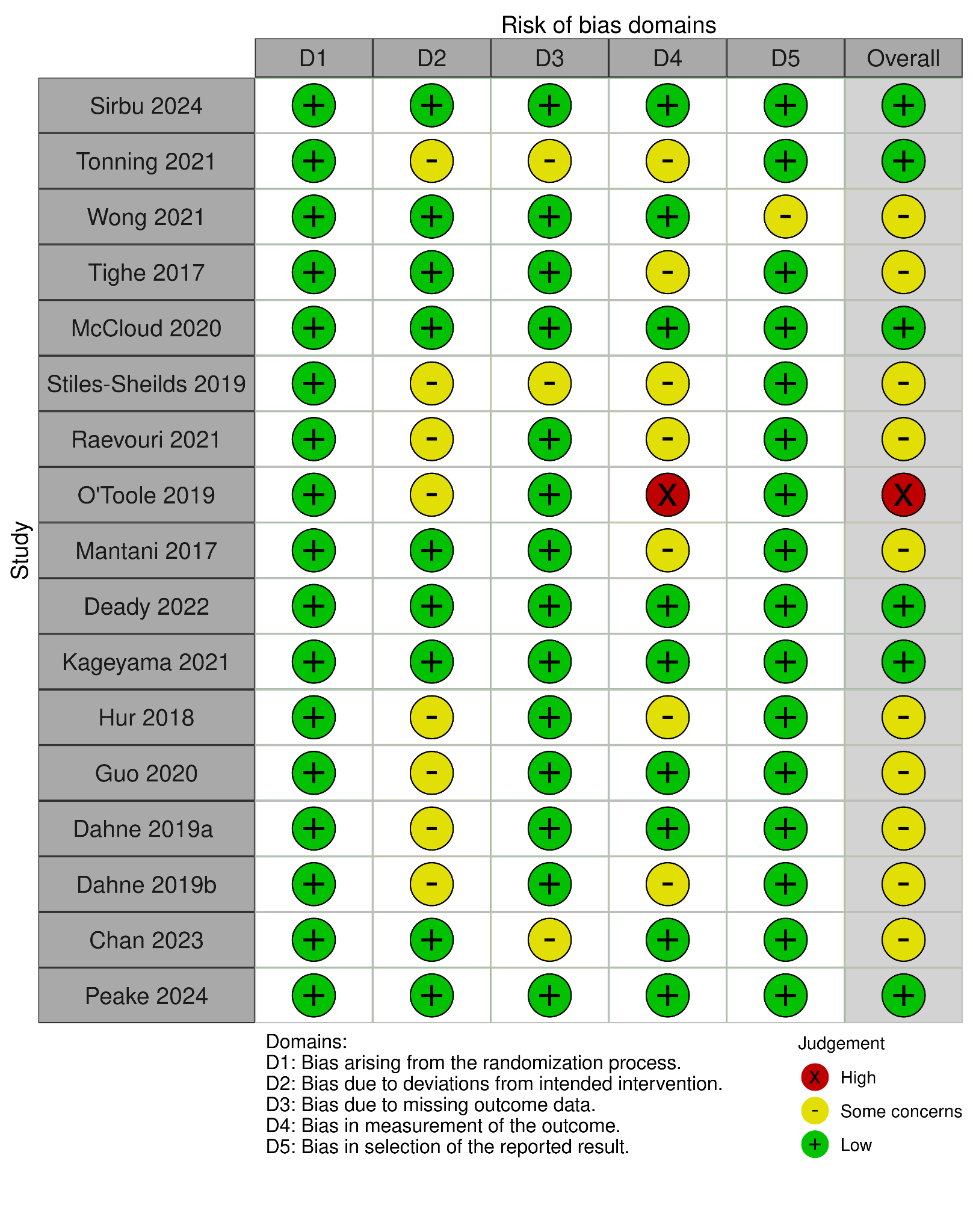
**

*Supp-Figure 10: Traffic Light Plot for Risk of Bias of Each Included Study*
